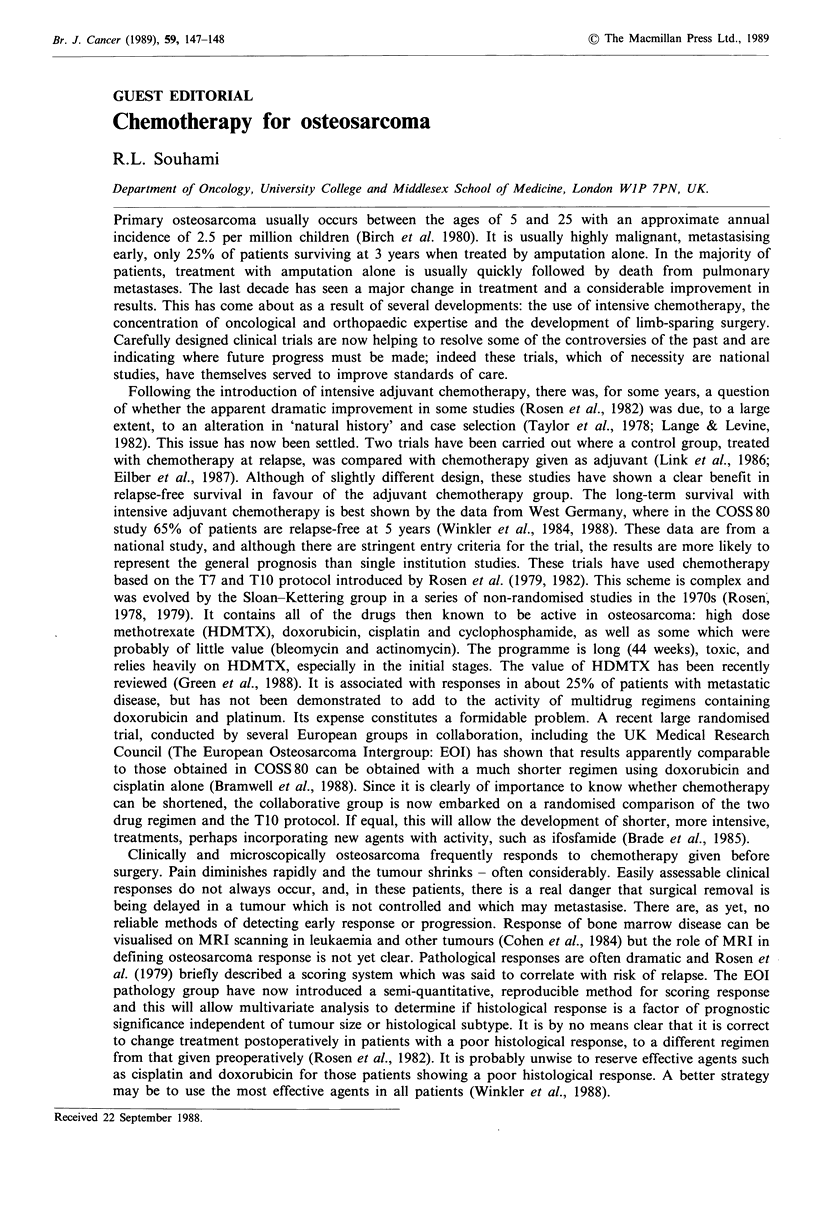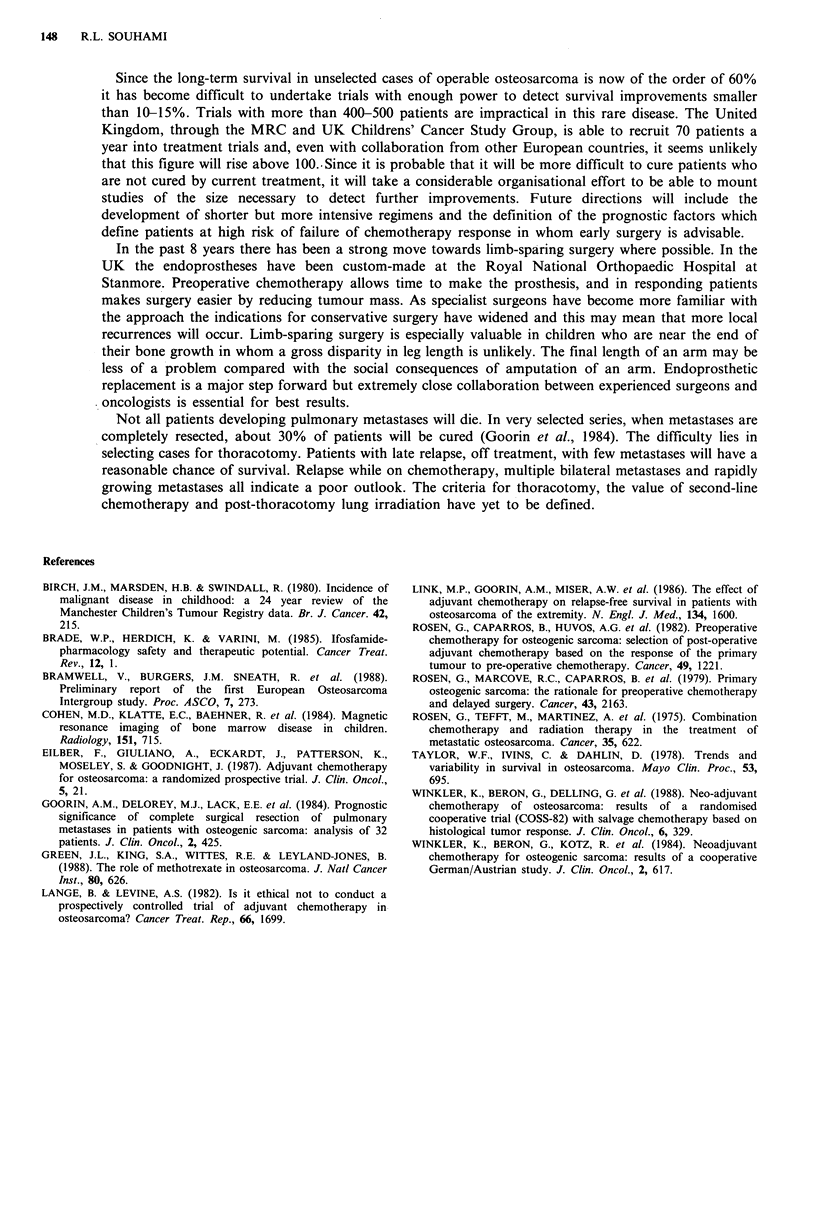# Chemotherapy for osteosarcoma.

**DOI:** 10.1038/bjc.1989.30

**Published:** 1989-02

**Authors:** R. L. Souhami

**Affiliations:** Department of Oncology, University College and Middlesex School of Medicine, London, UK.


*Br. J. Cancer* (1989), **59**, 147–148 © The Macmillan Press Ltd., 1989

GUEST EDITORIAL

### Chemotherapy for osteosarcoma

R.L. Souhami

*Department of Oncology, University College and Middlesex School of Medicine, London W1P 7PN, UK.*

Primary osteosarcoma usually occurs between the ages of 5 and 25 with an approximate annual incidence of 2.5 per million children (Birch *et al.* 1980). It is usually highly malignant, metastasising early, only 25% of patients surviving at 3 years when treated by amputation alone. In the majority of patients, treatment with amputation alone is usually quickly followed by death from pulmonary metastases. The last decade has seen a major change in treatment and a considerable improvement in results. This has come about as a result of several developments: the use of intensive chemotherapy, the concentration of oncological and orthopaedic expertise and the development of limb-sparing surgery. Carefully designed clinical trials are now helping to resolve some of the controversies of the past and are indicating where future progress must be made; indeed these trials, which of necessity are national studies, have themselves served to improve standards of care.

Following the introduction of intensive adjuvant chemotherapy, there was, for some years, a question of whether the apparent dramatic improvement in some studies (Rosen *et al.*, 1982) was due, to a large extent, to an alteration in 'natural history' and case selection (Taylor *et al.*, 1978; Lange & Levine, 1982). This issue has now been settled. Two trials have been carried out where a control group, treated with chemotherapy at relapse, was compared with chemotherapy given as adjuvant (Link *et al.*, 1986; Eilber *et al.*, 1987). Although of slightly different design, these studies have shown a clear benefit in relapse-free survival in favour of the adjuvant chemotherapy group. The long-term survival with intensive adjuvant chemotherapy is best shown by the data from West Germany, where in the COSS 80 study 65% of patients are relapse-free at 5 years (Winkler *et al.*, 1984, 1988). These data are from a national study, and although there are stringent entry criteria for the trial, the results are more likely to represent the general prognosis than single institution studies. These trials have used chemotherapy based on the T7 and T10 protocol introduced by Rosen *et al.* (1979, 1982). This scheme is complex and was evolved by the Sloan–Kettering group in a series of non-randomised studies in the 1970s (Rosen, 1978, 1979). It contains all of the drugs then known to be active in osteosarcoma: high dose methotrexate (HDMTX), doxorubicin, cisplatin and cyclophosphamide, as well as some which were probably of little value (bleomycin and actinomycin). The programme is long (44 weeks), toxic, and relies heavily on HDMTX, especially in the initial stages. The value of HDMTX has been recently reviewed (Green *et al.*, 1988). It is associated with responses in about 25% of patients with metastatic disease, but has not been demonstrated to add to the activity of multidrug regimens containing doxorubicin and platinum. Its expense constitutes a formidable problem. A recent large randomised trial, conducted by several European groups in collaboration, including the UK Medical Research Council (The European Osteosarcoma Intergroup: EOI) has shown that results apparently comparable to those obtained in COSS 80 can be obtained with a much shorter regimen using doxorubicin and cisplatin alone (Bramwell *et al.*, 1988). Since it is clearly of importance to know whether chemotherapy can be shortened, the collaborative group is now embarked on a randomised comparison of the two drug regimen and the T10 protocol. If equal, this will allow the development of shorter, more intensive, treatments, perhaps incorporating new agents with activity, such as ifosfamide (Brade *et al.*, 1985).

Clinically and microscopically osteosarcoma frequently responds to chemotherapy given before surgery. Pain diminishes rapidly and the tumour shrinks – often considerably. Easily assessable clinical responses do not always occur, and, in these patients, there is a real danger that surgical removal is being delayed in a tumour which is not controlled and which may metastasise. There are, as yet, no reliable methods of detecting early response or progression. Response of bone marrow disease can be visualised on MRI scanning in leukaemia and other tumours (Cohen *et al.*, 1984) but the role of MRI in defining osteosarcoma response is not yet clear. Pathological responses are often dramatic and Rosen *et al.* (1979) briefly described a scoring system which was said to correlate with risk of relapse. The EOI pathology group have now introduced a semi-quantitative, reproducible method for scoring response and this will allow multivariate analysis to determine if histological response is a factor of prognostic significance independent of tumour size or histological subtype. It is by no means clear that it is correct to change treatment postoperatively in patients with a poor histological response, to a different regimen from that given preoperatively (Rosen *et al.*, 1982). It is probably unwise to reserve effective agents such as cisplatin and doxorubicin for those patients showing a poor histological response. A better strategy may be to use the most effective agents in all patients (Winkler *et al.*, 1988).

Received 22 September 1988.

Since the long-term survival in unselected cases of operable osteosarcoma is now of the order of 60% it has become difficult to undertake trials with enough power to detect survival improvements smaller than 10–15%. Trials with more than 400–500 patients are impractical in this rare disease. The United Kingdom, through the MRC and UK Childrens' Cancer Study Group, is able to recruit 70 patients a year into treatment trials and, even with collaboration from other European countries, it seems unlikely that this figure will rise above 100. Since it is probable that it will be more difficult to cure patients who are not cured by current treatment, it will take a considerable organisational effort to be able to mount studies of the size necessary to detect further improvements. Future directions will include the development of shorter but more intensive regimens and the definition of the prognostic factors which define patients at high risk of failure of chemotherapy response in whom early surgery is advisable.

In the past 8 years there has been a strong move towards limb-sparing surgery where possible. In the UK the endoprostheses have been custom-made at the Royal National Orthopaedic Hospital at Stanmore. Preoperative chemotherapy allows time to make the prosthesis, and in responding patients makes surgery easier by reducing tumour mass. As specialist surgeons have become more familiar with the approach the indications for conservative surgery have widened and this may mean that more local recurrences will occur. Limb-sparing surgery is especially valuable in children who are near the end of their bone growth in whom a gross disparity in leg length is unlikely. The final length of an arm may be less of a problem compared with the social consequences of amputation of an arm. Endoprosthetic replacement is a major step forward but extremely close collaboration between experienced surgeons and oncologists is essential for best results.

Not all patients developing pulmonary metastases will die. In very selected series, when metastases are completely resected, about 30% of patients will be cured (Goorin *et al.*, 1984). The difficulty lies in selecting cases for thoracotomy. Patients with late relapse, off treatment, with few metastases will have a reasonable chance of survival. Relapse while on chemotherapy, multiple bilateral metastases and rapidly growing metastases all indicate a poor outlook. The criteria for thoracotomy, the value of second-line chemotherapy and post-thoracotomy lung irradiation have yet to be defined.

### References

BIRCH, J.M., MARSDEN, H.B. & SWINDALL, R. (1980). Incidence of malignant disease in childhood: a 24 year review of the Manchester Children's Tumour Registry data. *Br. J. Cancer.* **42,** 215.

BRADE, W.P., HERDICH, K. & VARINI, M. (1985). Ifosfamide-pharmacology safety and therapeutic potential. *Cancer Treat. Rev.*, **12,** 1.

BRAMWELL, V., BURGERS, J.M. SNEATH, R. *et al.* (1988). Preliminary report of the first European Osteosarcoma Intergroup study. *Proc. ASCO*, **7,** 273.

COHEN, M.D., KLATTE, E.C., BAEHNER, R. *et al.* (1984). Magnetic resonance imaging of bone marrow disease in children. *Radiology*, **151,** 715.

EILBER, F., GIULIANO, A., ECKARDT, J., PATTERSON, K., MOSELEY, S. & GOODNIGHT, J. (1987). Adjuvant chemotherapy for osteosarcoma: a randomized prospective trial. *J. Clin. Oncol.*, **5,** 21.

GOORIN, A.M., DELOREY, M.J., LACK, E.E. *et al.* (1984). Prognostic significance of complete surgical resection of pulmonary metastases in patients with osteogenic sarcoma: analysis of 32 patients. *J. Clin. Oncol.*, **2,** 425.

GREEN, J.L., KING, S.A., WITTES, R.E. & LEYLAND-JONES, B. (1988). The role of methotrexate in osteosarcoma. *J. Natl Cancer Inst.*, **80,** 626.

LANGE, B. & LEVINE, A.S. (1982). Is it ethical not to conduct a prospectively controlled trial of adjuvant chemotherapy in osteosarcoma? *Cancer Treat. Rep.*, **66,** 1699.

LINK, M.P., GOORIN, A.M., MISER, A.W. *et al.* (1986). The effect of adjuvant chemotherapy on relapse-free survival in patients with osteosarcoma of the extremity. *N. Engl. J. Med.*, **134,** 1600.

ROSEN, G., CAPARROS, B., HUVOS, A.G. *et al.* (1982). Preoperative chemotherapy for osteogenic sarcoma: selection of post-operative adjuvant chemotherapy based on the response of the primary tumour to pre-operative chemotherapy. *Cancer*, **49,** 1221.

ROSEN, G., MARCOVE, R.C., CAPARROS, B. *et al.* (1979). Primary osteogenic sarcoma: the rationale for preoperative chemotherapy and delayed surgery. *Cancer*, **43,** 2163.

ROSEN, G., TEFFT, M., MARTINEZ, A. *et al.* (1975). Combination chemotherapy and radiation therapy in the treatment of metastatic osteosarcoma. *Cancer*, **35,** 622.

TAYLOR, W.F., IVINS, C. & DAHLIN, D. (1978). Trends and variability in survival in osteosarcoma. *Mayo Clin. Proc.*, **53,** 695.

WINKLER, K., BERON, G., DELLING, G. *et al.* (1988). Neo-adjuvant chemotherapy of osteosarcoma: results of a randomised cooperative trial (COSS-82) with salvage chemotherapy based on histological tumor response. *J. Clin. Oncol.*, **6,** 329.

WINKLER, K., BERON, G., KOTZ, R. *et al.* (1984). Neoadjuvant chemotherapy for osteogenic sarcoma: results of a cooperative German/Austrian study. *J. Clin. Oncol.*, **2,** 617.